# Evaluation of Navigated Laser Photocoagulation (Navilas 577+) for the Treatment of Refractory Diabetic Macular Edema

**DOI:** 10.1155/2018/3978514

**Published:** 2018-05-02

**Authors:** Fusae Kato, Miho Nozaki, Aki Kato, Norio Hasegawa, Hiroshi Morita, Munenori Yoshida, Yuichiro Ogura

**Affiliations:** Department of Ophthalmology and Visual Science, Nagoya City University Graduate School of Medical Sciences, 1-Kawasumi, Mizuho-ku, Nagoya 467-8601, Japan

## Abstract

**Purpose:**

To evaluate navigated laser photocoagulation for the treatment of refractory diabetic macular edema (DME).

**Methods:**

Retrospective study of 25 eyes (21 patients) treated with Navilas 577+ focal laser system. Best-corrected visual acuity (BCVA) and spectral-domain optical coherence tomography (OCT) parameters were measured at baseline, 1, 3, and 6 months, and final visit.

**Results:**

The mean follow-up period was 12.8 ± 2.4 (7–16 months). All subjects had history of previous treatment which was injection of triamcinolone acetonide or antivascular endothelial growth factor (VEGF) agents. The navigated laser photocoagulation was delivered to the microaneurysms on indocyanine green angiography (ICGA) in 21 of 25 eyes (84%), fluorescein angiography (FA) guided in 3 eyes, and OCT angiography guided in 1 eye. After initial navigated laser treatment, 16 of 25 eyes (64%) were needed additional navigated laser photocoagulation, injection of triamcinolone acetonide, and/or injection of VEGF agents. Although median BCVA remained stable, the central retinal thickness and macular volume were significantly decreased over 6 months (*p* < 0.05). All patients were treated without complications.

**Conclusions:**

Focal photocoagulation using Navilas 577+ showed to be effective in treating DME with improvement in macular edema on OCT over 6 months. Navilas 577+ was beneficial to perform navigated laser photocoagulation based on three modalities (ICGA, FA, and OCT angiography).

## 1. Introduction

Diabetic macular edema (DME) occurs due to a malfunction of the blood-retinal barrier and death of endothelial cells leading to leakage of fluid and subsequent photoreceptor dysfunction [1, 2]. Focal laser treatment leads to the occlusion of these leaking microaneurysms (MAs), pathologic vessels, or subretinal sites of leakage [3]. As the Early Treatment Diabetic Retinopathy Study (ETDRS) research group showed, focal laser therapy can reduce moderate to severe vision loss, but the major effects were not seen till after 3 years of follow-up [4]. On the other hand, the advent of antivascular endothelial growth factor (VEGF) agents showed rapid and prominent effects on vision improvement in numerous multicenter trials [5–9]. Anti-VEGF agents have become the first-line treatment for DME. However, this treatment requires repeated intravitreous injections for an indefinite period, and safety concerns regarding to long-term systemic suppression of VEGF, which is a serious risk of cerebrovascular accidents especially in elderly patients, are emerging [10]. Recent meta-analysis has shown type 2 diabetic patients with DME or proliferative diabetic retinopathy (PDR) were associated with a twofold higher risk of fatal cardiovascular accidents compared with those without DME or PDR [11]. Therefore, a new optical treatment modality should be developed to improve the cost-effectiveness, safety, and visual outcomes. Liegl et al. reported the efficacy of a standardized combination therapy regimen (three ranibizumab injections followed by navigated focal laser) [12]. In their analysis, combination therapy regimen was significantly lower compared to ranibizumab monotherapy in terms of retreatment rate and number of injections among 12 months.

The navigated laser photocoagulator, also known as the Navilas laser system (OD-OS GmbH, Teltow, Germany), is computer-based system combined with wide-angle imaging camera. The Navilas has eye-tracking laser delivery system and allows more accurate for focal laser photocoagulation than conventional focal laser therapy for DME [13, 14]. The Navilas laser photocoagulation is performed based on preplanned treatment locations with the real-time fundus image. Navilas 577+ laser system is the new model of navigated laser system and has been approved in Japan in 2016. The preplanned treatment can be made based on color image, fluorescein angiography (FA), indocyanine green angiography (ICGA), optical coherence tomography (OCT), and/or OCT-angiography.

The aim of this study is to evaluate navigation laser photocoagulation (Navilas 577+ laser system) for the treatment of refractory DME patients.

## 2. Materials and Methods

This study was a retrospective, noncomparative case series performed at the eye center of Nagoya City University Graduate School of Medical Sciences. Institutional Review Board (IRB) approval (#60-17-0108) was obtained for the study protocol and procedures. The study adhered to the tenets of the Declaration of Helsinki.

Twenty-five eyes of 21 patients (14 men and 7women) with DME were included in this study between April 2016 and October 2016. The mean age was 68.3 ± 9.2 years (range 42–80 years). The mean follow-up period was 12.8 ± 2.4 (7–16 months). Before navigated laser treatment, 13 of 25 eyes (52%) had received sub-Tenon's injections of triamcinolone acetonide (TA) (Kenacort; Bristol-Myeres Squibb, Tokyo, Japan) and 12 or 25 eyes (48%) had received ranibizumab and/or aflibercept injections. Four eyes had received subthreshold laser using PASCAL Streamline (Topcon Medical Laser Systems, Santa Clara, CA, USA), and three eyes had received manual focal laser. One eye had received vitrectomy. There were some that overlapped in treatment history (Table 1).

All participants underwent a complete ophthalmologic examination, including the best-corrected visual acuity (BCVA), intraocular pressure, slit-lamp and indirect ophthalmoscopy, and OCT. The BCVA was measured with a Japanese standard decimal visual acuity chart, and decimal BCVA was calculated using the logarithm of the minimum angle of resolution (LogMAR) scale. Spectral-domain OCT (Cirrus HD-OCT; Carl Zeiss Meditec, Dublin, CA) was performed to evaluate morphological retinal changes, central retinal thickness (CRT), and macular volume (MV). FA and ICGA using the Heidelberg Spectralis HRA II (Heidelberg Engineering, Heidelberg, Germany) were performed to detect the leakage of MAs. DME type was classified as focal or diffuse based on the features below. The characteristics of focal macular edema are (1) location outside the foveal center with or without center involvement; (2) asymmetric increases in retinal thickness on OCT scan; and (3) accumulation of pin-point leakage in early phase. The characteristics of diffuse macular edema are (1) increased retinal thickness with center involvement on the OCT macular thickness map; (2) symmetrically increased retinal thickness on B-scan OCT; and (3) fluorescein leakage starting from early phase and continuously increasing to late phase [2]. Fifteen eyes (60%) were classified as focal and 10 eyes (40%) were classified as diffuse edema.

A yellow wavelength (577 nm) photocoagulation was planned to target the leaking MAs and performed by the Navilas laser system. Briefly, treatment plan was made by physicians on a static image from FA, ICGA, and/or OCTA. The image which registered and overlaid onto the live retinal image in real time was on placing laser spot marks. After photocoagulation, a color image of the fundus was acquired to confirm that all laser applications accurately hit the preplanned points. In follow-up examinations, the patients received additional photocoagulation or medications if there were persistent of MAs or there were no findings of reduction in CRT.

Statistical analyses were performed with SPSS statistics 22 (IBM Corp., Armonk, NY, USA). In evaluating BCVA, CRT, and MV, changes (1, 3, 6 months and final visit) from baseline were analyzed using one-way repeated measures ANOVA and Bonferroni correction as post hoc test. A *p* value of <0.05 was considered statistically significant.

## 3. Results

The initial navigated laser photocoagulation was delivered to the MAs on ICGA in 21 of 25 eyes (84%). Three eyes underwent FA-guided photocoagulation. One eye had the allergy of dye injection and had undergone navigated photocoagulation on OCT angiography (Avanti OCT; Optovue, Fremont, CA, USA). The mean laser parameters were a spot size of 50–100 *μ*m, duration between 20–100 milliseconds (ms), and power between 50–100 milliwatts (mW). Each patient received an average of 22 ± 17 burns for successful treatment. The initial laser application was performed without a contact lens in 12 of 25 eyes (48%).

After initial Navilas laser treatment, 9 of 25 eyes (36%) did not need any retreatments due to resolution of DME, although 4 eyes received sub-Tenon's injections of TA simultaneously. In other subjects, macular edema remained. Two eyes received intravitreal aflibercept (IVA) prior to navigated laser treatment within 4 weeks and 9 eyes were received IVA after navigated laser treatment. The average number of IVA is 1.55 ± 1.00 between 6 months. Some eyes received sub-Tenon' injections of TA and/or intravitreous injection of TA (MaQaid; Wakamoto Pharmaceutical Co., Ltd., Tokyo, Japan). Eleven eyes (44%) carried out additional Navilas laser photocoagulation. Three eyes were performed subthreshold laser, and 2 eyes were performed focal laser treatment by the slit-lamp delivery laser system (PASCAL Streamline) (Table 2).

**Figure 1:**
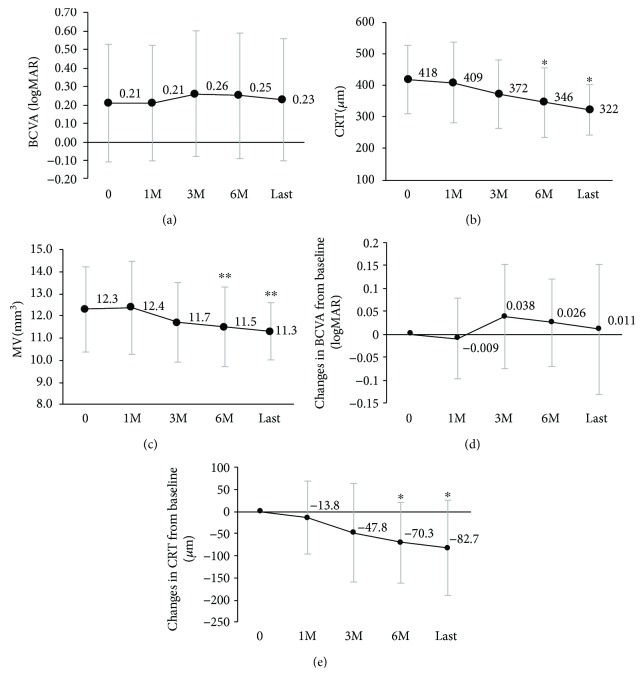
Comparison of pretreatment and posttreatment of the BCVA (a, d), CRT (b, e), and MV (c). The mean VA (± standard deviation) was unchanged from baseline to final visit; CRT and MV improved significantly (^∗^*p* < 0.05, ^∗∗^*p* < 0.05). The comparison with baseline was evaluated by means of Bonferroni adjustment.

Changes of mean visual acuity and parameters of OCT were showed in Figure 1. Mean logMAR BCVA, CRT, and MV at baseline were 0.21 ± 0.32, 417.7 ± 108.3 *μ*m, and 12.3 ± 1.9 mm^3^, respectively. Mean CRT at 6 months and the final visit decreased significantly compared with baseline (month 6; 346.4 ± 110.4 *μ*m, the final visit; 322.3 ± 78.6 *μ*m, *p* < 0.05). Mean MV at 6 months and the final visit also decreased significantly compared with baseline (month 6; 11.5 ± 1.8 mm^3^, final visit; 11.3 ± 1.3 mm^3^, *p* < 0.01). There were no remarkable changes in mean LogMAR BCVA after the navigated laser treatment (month 6; 0.25 ± 0.34, final visit; 0.23 ± 0.33). Eight eyes (89%) of nonretreated group were focal DME, whereas seven eyes (44%) of retreated group were focal edema. The difference in the morphology of DME between the nonretreated and retreated groups was significant (*p* < 0.05, fisher's exact test). There were no complications related to laser treatment.

### 3.1. Representative Cases of ICGA-Guided Navilas 577+ Laser Photocoagulation

**Figure 2:**
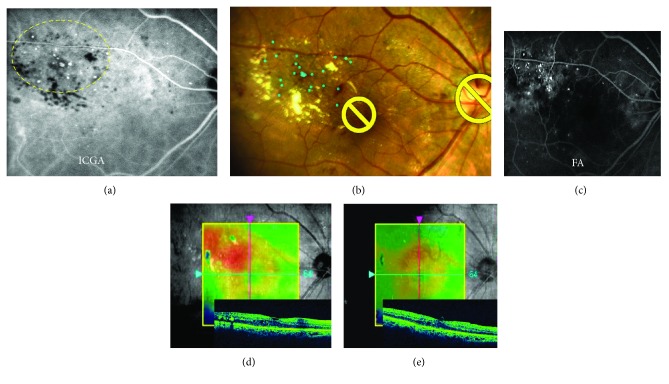
Representative case of ICGA-guided Navilas 577+ laser photocoagulation. A 77-year-old man underwent ICGA-guided navigated laser photocoagulation to treat DME which remained after focal laser using PASCAL (a–d). Image of treatment plan (blue dots) (b) was based on ICGA (a). After the navigated laser treatment, the macular edema was decreased in 3 months with no recurrences (e). The decimal visual acuity remained 0.9.

A 77-year-old man presented with vision deterioration of the right eye due to DME. OCT macular map image revealed fovea-involving macular edema, and leaking MAs were detected on FA and ICGA corresponding with the OCT thickness map findings. Although combined therapy with sub-Tenon's capsular injection of TA and ICGA-guided manual focal laser photocoagulation using PASCAL had been applied in March 2016, the macular edema remained a month later. The navigated laser photocoagulation was planned and performed based on ICGA in April 2016. There was resolution of DME in 3 months after the navigated laser treatment (Figures 2(a)–2(e)).

**Figure 3:**
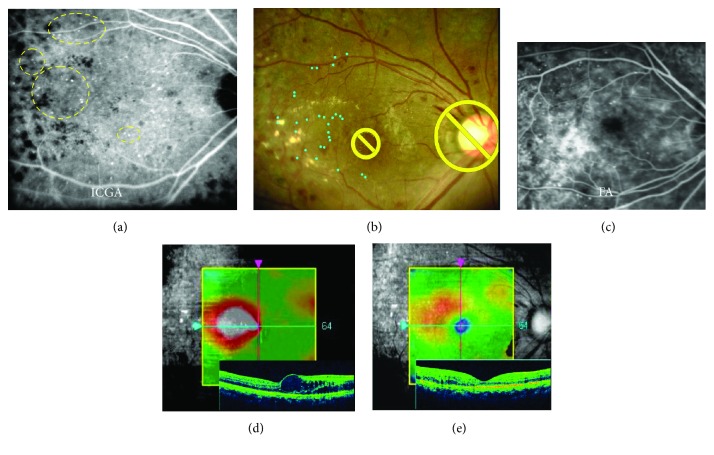
Representative case of ICGA-guided Navilas 577+ laser photocoagulation. A 42-year-old female underwent ICGA-guided navigated laser therapy (a–e). Some MAs were detected on late-phase ICGA indicated by yellow dashed line (a). Early-phase FA showed diffuse leakage from numerous MAs (c). Image of treatment plan (blue dots) (b) was based on ICGA (a). One month after the navigated laser photocoagulation, OCT findings on macular map and cross-sectional images became thinner and decimal visual acuity was remarkably improved from 0.5 to 0.8 (e).

A 42-year-old female consulted to our hospital with a complaint of vision deterioration in the right eye, which was affected with DME developed after panretinal photocoagulation. The eye had the history of steroid-induced glaucoma. ICGA-guided Navilas laser photocoagulation was planned and underwent in April 2016. One month after the navigated laser photocoagulation, OCT findings on macular map and cross-sectional images became thinner and decimal visual acuity was remarkably improved from 0.5 to 0.8 (Figures 3(a)–3(e)).

## 4. Discussion

This retrospective case series of eyes with DME treated by Navilas 577+ laser system demonstrated reduction in CRT and MV at 6 months and final visit but no significant difference in logMAR BCVA. Moreover, other recent studies related to combination therapy of navigated laser and anti-VEGF agent have been published [12, 15, 16]. Their results indicate combination therapy is effective for visual gain and retinal stabilization, which can reduce number of injections. In our study, 11 eyes received injection of aflibercept following initial navigated laser, and their number of injection for 6 months were 1.55 ± 1.0, which was fewer than patients in major clinical trials [17]. As references, DME patients (23 eyes) treated anti-VEGF monotherapy in our institution received 2.52 ± 1.0 injections for the same duration of time. We considered there were two reasons why significant visual gain was not found in our study. One reason was many subjects had received several treatment histories before navigated focal laser was applied. The other was baseline BCVA in the current study was better than in those previous studies.

A randomized trial with navigated laser therapy (TREX-DME) for DME did not detect therapeutic benefits for navigated laser photocoagulation in visual gain and the CRT improved. The number of injections was not also significantly reduced at one year in combination therapy with anti-VEGF and navigated laser photocoagulation [18]. Although there was no significant difference in the mean maximal treatment interval with or without navigated laser photocoagulation, 38% of eyes with navigated laser photocoagulation were able to be maximally extended to 12 weeks, the benefit of adding navigated laser photocoagulation might be obvious with longer-term follow-up [18].

Predominantly, focal leakage from MAs showed less responsive to anti-VEGF therapy [19]. In our study, especially in cases where leaking MAs are mainly localized outside of the perifoveal capillary network, navigated laser therapy was effective. Other studies demonstrated combined conventional focal laser treatment could reduce the number of anti-VEGF injections for focal DME [7, 20]. To detect efficacy of focal laser therapy, it might be important individualized treatment be classified with different leakage subtype.

To mention with distinctive features in our current study, ICGA-guide navigated laser was performed to most of study eyes (84%). Indocyanine green dye is 98% bound to lipoproteins in the blood. Therefore, the dye hardly leaks, ICGA defines the detailed retinal vascular abnormalities better than FA [21–24]. Previously, we have reported that middle- to late-phase ICGA images show responsible MAs adjacent retinal edema, resulting in more precise and less number of focal laser photocoagulation spots [25, 26], and other groups also reported the clinical efficacy of ICGA-guided laser [27, 28]. However, it is difficult to identify the location of MAs on ICGA, due to lack of information of foveal avascular zone. So, the navigated laser system overlaid fundus image is suitable for treatment with ICGA-guided laser photocoagulation. The navigated photocoagulation seems to demonstrate a higher laser spot application accuracy in focal laser therapy of DME than conventional laser technique [13, 14]. However, in our study, 44% of eyes were required additional navigated laser photocoagulation. The result may mean that it takes time to become skilled in performing laser photocoagulation with navigation system. Navilas laser system enables physician to coagulate MA under observing the fundus directly. Especially conventional laser photocoagulation for MA, the aiming beam has been focused on forward to the retinal pigment epithelium (RPE). With Navilas, the location in the X-Y directions is accurate, but the focus in the *z*-axis is impossible to adjust. Boiko and Maltsev reported the larger diameter of laser burns and the more laser power needed following navigated focal laser in edematous retina compared with dry retina [29]. Therefore, it is recommended that navigated laser photocoagulation is performed under dry retinal conditions following a combination of intravitreal anti-VEGF or steroid injection with prompt or deferred focal laser treatment. In our study, 6 of 25 eyes (24%) received intravitreal anti-VEGF or steroid injection with prompt navigated focal laser treatment. Although there was no significant difference in retreatment rate of navigated laser photocoagulation with or without pretreatment of pharmacotherapy, it might be important to establish ideal protocol for treating thickened macular edema by Navilas laser system in future.

In addition, the MAs associated with DME were mainly found in deep capillary plexus of retina based on the OCT angiography (OCTA) [30]. Although OCTA cannot be used to visualize leakage, it is noninvasive, nondye imaging modality. In our study, only one eye was treated with OCTA-guided NAVILAS focal laser for the MAs located in deep capillary plexus, and we hope to study more number of eyes with OCTA-guided navigated focal laser in future.

There are several limitations to our current study. Because this study was a retrospective study, the additional intervention protocols, which were additional laser photocoagulation, anti-VEGF therapy, or steroid therapy, were not determined. This study was a nonrandomized study with no control groups and had relatively small number of patients and short follow-up period. Larger number and longer follow-up study would be warranted to study the efficacy of navigated focal laser photocoagulation for DME in future.

## 5. Conclusions

In conclusion, our study shows a significant decreasing of macular thickness using navigated laser photocoagulation based on multimodal imaging.

## Figures and Tables

**Table 1:** Baseline characteristics of patients.

Characteristic	Value
Number of eyes/patients	25 eyes/21 patients
Sex, *n* (%)	
Male	14 (64%)
Female	7 (36%)
Age (years)	
Range	42–80
Mean ± SD	68.3 ± 9.2
HbA1C (%)	
Mean ± SD	7.1 ± 1.2
Previous treatments, *n*	
STTA	13^∗^
Anti-VEGF (ranibizumab, aflibercept)	12^∗^
Subthreshold laser	4^∗^
Conventional focal laser	3^∗^
Vitrectomy	1^∗^

^∗^Including the overlap; VEGF: vascular endothelial growth factor; STTA: sub-Tenon's injections of triamcinolone acetonide.

**Table 2:** The details of additional treatment after initial navigated laser photocoagulation.

No retreatment, *n* (%)	9 (36%)
Single navigated laser	5 (20%)
Navigated laser + STTA	4 (16%)
Additional treatment	16 (64%)
IVA	11^∗^
STTA	6^∗^
IVTA	5^∗^
Navigated laser	11^∗^
Subthreshold laser	3^∗^
Manual focal laser	2^∗^

^∗^Including the overlap; STTA: sub-Tenon's injections of triamcinolone acetonide; IVA: intravitreal aflibercept; IVTA: intravitreal triamcinolone acetonide.
